# Knowledge, Attitude, and Practice Regarding Child Abuse Among Healthcare Workers in Al Dhahira, Oman: A Cross-Sectional Study

**DOI:** 10.7759/cureus.113915

**Published:** 2026-08-03

**Authors:** Noora A Al Sukaiti

**Affiliations:** 1 Emergency Department, Ibri Hospital, Ibri, OMN

**Keywords:** abuse reporting, attitude, child abuse, child protection, healthcare workers, knowledge, oman, practice, primary health care, reporting barriers

## Abstract

Background: Child abuse remains a significant public health challenge. Healthcare workers serve as critical frontline responders, yet gaps persist between legislative frameworks and implementation.

Objective: The objective of this study is to assess knowledge, attitudes, and practices of healthcare workers in Al Dhahira regarding child abuse identification and reporting, identify barriers to consistent implementation, and examine factors associated with reporting behavior.

Methods: A descriptive cross-sectional study of 150 healthcare workers at 18 primary healthcare centers in Al Dhahira was conducted in 2023 (sample size calculated as 148 for a population of 600, 95% confidence level). Data were analyzed using IBM SPSS Statistics for Windows, Version 25 (Released 2017; IBM Corp., Armonk, New York, United States) with descriptive statistics and chi-square tests (p<0.05).

Results: The sample comprised 102 (68.0%) physicians, 38 (25.3%) nurses, and 10 (6.7%) allied health professionals. While 140 (93.3%) were aware of the Omani Child Law and 128 (85.3%) knew a child protection hotline exists, only 110 (73.3%) knew the correct hotline number; 91 of 128 (71.1%) among those aware. Although 138 (92.0%) believed suspected abuse should always be reported, only 110 (73.3%) consistently reported cases, revealing an 18.7-percentage-point attitude-practice gap. Major barriers included fear of legal consequences (44; 29.3%), clinical identification challenges (42; 28.0%), and lack of feedback (39; 26.0%). Previous child protection training was significantly associated with consistent reporting behavior: 73 of 89 (82.0%) trained workers consistently reported versus 37 of 61 (60.7%) untrained workers (p=0.001).

Conclusions: Healthcare workers demonstrate good theoretical knowledge of child protection frameworks but face significant practical barriers to consistent implementation. Evidence-based interventions including professional development programs, clear legal protection communications, feedback mechanisms, and multi-professional reporting capacity warrant prospective evaluation to bridge the attitude-practice gap and ensure effective child protection implementation.

## Introduction

Child abuse represents a major global health emergency, with approximately one billion children aged 2-17 years experiencing violence annually, particularly in the Eastern Mediterranean Region [1]. The Sultanate of Oman has developed a comprehensive child protection framework through Royal Decree No. 22/2014, which mandates reporting of suspected abuse and establishes a national hotline (1100) [2]. Healthcare workers, including physicians, nurses, paramedical staff, and allied health professionals, stand at the frontlines of child protection. Community health workers in primary healthcare settings play a critical role, though their knowledge, attitudes, and practices regarding child abuse identification vary across settings [3].

Significant gaps persist between what healthcare workers know they should do and what they actually do. Nurses face multiple barriers to reporting, including fear of legal consequences and clinical identification challenges [4], while emergency department staff encounter distinctive barriers related to high-volume patient loads [5]. Research from the Middle East demonstrates that nurses' intentions to report child maltreatment are influenced by multiple factors [6].

Oman's child protection capacities have evolved significantly [7], though systematic evaluation reveals gaps in implementation capacity despite high readiness in legislation [8]. Continuous, multidisciplinary training improves healthcare professional recognition and reporting of child abuse [9]. Oman's commitment to a public health approach benchmarking against the INSPIRE Strategy provides a strategic framework [10]. Studies from diverse healthcare settings demonstrate substantial variations in reporting behavior despite high willingness to report; for example, research among Saudi physicians shows that fewer than one in seven report suspected cases in clinical practice despite over 90% expressing willingness to report [11]. Qualitative research exploring how primary healthcare professionals recognize suspected abuse has identified that identification often relies on subtle clinical cues and intuitive judgment rather than formal diagnostic criteria alone [12]. A comprehensive scoping review across Arab Gulf Cooperation Council countries identified 160 articles on child maltreatment; however, intervention research remains lacking, and Oman contributed only 12 of these articles [13].

Al Dhahira region, located in Oman's interior with 18 primary healthcare centers serving sparsely populated areas, presents distinct implementation challenges where remote primary healthcare centers directly affect child protection capabilities. In remote communities, primary healthcare centers serve as the sole point of medical contact, concentrating child protection responsibilities on small teams managing all clinical functions. These workers operate without specialist access, immediate consultation resources, or on-site child protection focal persons, factors that limit capacity for complex case management and increase delays in recognition and reporting. The physical distance from specialized services limits coordination and follow-up on reported cases. Limited local expertise and training resources may result in lower knowledge levels and fewer opportunities for professional development in child abuse recognition, potentially reducing identification and reporting rates compared to urban settings. These factors collectively suggest that healthcare worker knowledge, attitudes, and practices regarding child abuse may differ substantially from urban and coastal settings and regional benchmarks.

Within Oman, no systematic assessment has characterized healthcare worker knowledge, attitudes, and practices in remote primary healthcare settings such as Al Dhahira. Understanding how geographic remoteness and limited resources affect child protection capabilities is essential to develop targeted interventions appropriate to Al Dhahira's circumstances and to provide insights for similar settings across Oman and the Gulf Cooperation Council region.

Study aim and objectives

This study aimed to assess knowledge, attitudes, and self-reported practices of healthcare workers in Al Dhahira region regarding child abuse identification and reporting. To achieve this primary aim, the study had four specific objectives: (i) to describe healthcare workers' knowledge of the Omani Child Protection Law, legal definitions of child abuse, and awareness of reporting mechanisms including the national child protection hotline; (ii) to assess attitudes toward mandatory reporting of suspected child abuse; (iii) to identify specific barriers and enablers to consistent implementation of the Omani Child Law in primary healthcare settings; and (iv) to examine factors associated with consistent reporting behavior, including prior child protection training participation, professional role, years of experience, and knowledge level.

## Materials and methods

Study design and setting

A descriptive cross-sectional study was conducted among healthcare workers employed at primary healthcare centres in Al Dhahira region during 2023. The region includes three wilayats (administrative divisions): Ibri, Yanqul, and Dhank, with 18 primary healthcare centers serving a population of approximately 250,000 residents.

Study population and sampling

From a total workforce of 600 healthcare workers across 18 primary healthcare centres in Al Dhahira, all healthcare workers meeting predefined inclusion criteria were invited to participate using a census-invitation approach. Inclusion criteria were (i) current employment with primary healthcare services in Al Dhahira, (ii) direct patient care responsibilities, and (iii) willingness to participate. Exclusion criteria were (i) administrative staff without clinical experience and (ii) temporary or contract workers with <6 months of employment. The survey was distributed through the regional health directorate's official communication channels to all eligible healthcare workers. A total of 154 responses were received, of which 150 complete questionnaires were analyzed, representing a 97.4% completion rate among returned questionnaires. Because participation was voluntary, the final sample represented a self-selected respondent sample rather than a complete census, and self-selection or non-response bias cannot be excluded.

Data collection instrument

Questionnaire Development and Validation

The questionnaire was developed based on study objectives and a comprehensive review of literature on child abuse knowledge, attitudes, and practices in healthcare settings. Items were designed to assess four key domains: (i) knowledge of child protection legislation and reporting procedures, (ii) awareness of reporting mechanisms, (iii) attitudes toward mandatory reporting, and (iv) self-reported reporting practices and barriers to implementation.

The questionnaire underwent expert panel review by the research team and members of the Governorate Child Protection Committee to assess items for clarity, relevance, and appropriateness within the Al Dhahira healthcare context. This expert review supported face and content relevance, though formal quantitative assessment (e.g., Content Validity Index) was not conducted, as the instrument was designed for descriptive assessment in this specific setting rather than as a validated measurement tool requiring psychometric rigor for broader application.

Pilot testing with 15 healthcare workers (physicians, nurses, and allied health professionals) from Al Dhahira primary healthcare centres provided feedback on clarity and comprehensibility. Two items assessing knowledge of reporting mechanisms and hotline awareness required wording simplification; these were refined based on pilot feedback. The questionnaire demonstrated good overall comprehensibility with no items requiring substantial restructuring, and average completion time was 8-10 minutes.

Formal reliability testing (Cronbach's alpha) was not conducted. As the questionnaire comprises heterogeneous items assessing distinct constructs (knowledge, attitudes, practices, barriers) rather than a single latent construct, and several domains were assessed using individual items, Cronbach’s alpha was not appropriate for the overall questionnaire. Expert review and pilot testing were used to assess item clarity, content relevance, comprehensibility, and feasibility rather than statistical reliability.

Questionnaire Administration and Data Collection (2023)

The refined questionnaire was administered electronically via Google Forms during a 2023 quality improvement and audit activity. The questionnaire link was distributed by unit and departmental in-charges to healthcare workers meeting inclusion criteria (current employment in direct patient care roles at Al Dhahira primary healthcare centres, willingness to participate). The questionnaire was self-administered in English, allowing healthcare workers to complete it independently with flexible completion timing. Informed consent was obtained verbally from all participants prior to questionnaire completion. Participation was entirely voluntary.

Participants were instructed to complete the questionnaire only once. Email addresses provided for verification purposes were cross-referenced against response data to ensure single participation per individual; no duplicate responses were identified during data collection. Responses with any unanswered items were considered incomplete and excluded from analysis. Of 154 responses received, 150 met completeness criteria and were included in the final analysis.

Email addresses were collected solely to verify unique participation and were removed before data analysis. The analytical dataset was therefore completely de-identified, with no identifying information linked to participants' substantive responses. All responses were kept secure throughout data collection and analysis, with all data maintained confidentially.

Subsequent Research Analysis (2025)

Following ethical approval obtained in 2025 from the Research and Studies Committee of the Directorate General of Health Services, Al-Dhahira Governorate (Approval number: MH/DGHS/DG 2623183342/25, approved 30 November 2025), the 2023 audit data were formally analyzed as a descriptive cross-sectional research study.

Statistical analysis

Data were analyzed using IBM SPSS Statistics for Windows, Version 25 (Released 2017; IBM Corp., Armonk, New York, United States) with descriptive statistics (frequencies, percentages) for all variables.

Score categorization and cut-off values

Knowledge Assessment

Four knowledge items (awareness of Omani Child Law, legal definition of child <18 years, existence of national hotline, and correct hotline number 1100) were scored dichotomously as correct or incorrect. "Not sure" responses were scored as incorrect. A total knowledge score was calculated as percentage of correct responses (0-100%). Knowledge was categorized as High (>80%) and Low (≤80%). The >80% threshold represents the standard educational competency benchmark in healthcare professional training, with research demonstrating that >80% knowledge of reporting procedures is associated with consistent reporting behavior and appropriate clinical practice.

Attitudes and Practices

Attitudes toward mandatory reporting ("Cases of suspected child abuse should be notified") were dichotomized as Supportive (Always) versus Non-supportive (Often/Occasionally/Never), reflecting the binary nature of mandatory reporting. Self-reported reporting practices ("When I encountered cases with suspected child abuse, they were notified") were dichotomized as Consistent (Always) versus Inconsistent (Usually/Sometimes/Never), as only those reporting "Always" demonstrate consistent compliance with mandatory requirements. Awareness of institutional systems (clinical guidelines, responsibility for e-notification) was analyzed descriptively.

Barriers and Training

Barriers to reporting were assessed using a single item with four mutually exclusive response options: inability to clinically identify child abuse cases, lack of identified focal person, fear of legal consequences, and lack of feedback about patient outcomes. Participants selected one option representing their primary barrier, analyzed descriptively by frequency. Prior training participation was dichotomized as Trained (attended training more than once or once) versus Untrained (never attended). Participants with any training experience were categorized as trained to distinguish those with exposure to child protection education from those with no training exposure.

Demographic Variables

Years of professional experience was categorized as <5 years, 5-10 years, and >10 years, consistent with questionnaire response options and reflecting clinically meaningful career progression stages from novice to experienced practitioners.

Statistical testing

Chi-square tests examined associations between demographic characteristics and categorical variables (knowledge level, attitudes, practices, training status). Fisher's exact test was applied when expected cell counts were less than 5. Statistical significance was set at p<0.05.

## Results

Demographic characteristics

A total of 150 healthcare workers from 18 primary healthcare centers completed the survey. The sample comprised 102 (68.0%) physicians, 38 (25.3%) nurses, and 10 (6.7%) other allied health professionals. Years of experience showed the following: 45 (30.0%) with less than five years, 58 (38.7%) with 5-10 years, and 47 (31.3%) with more than 10 years of clinical experience, reflecting three clinically meaningful career progression stages from novice to experienced practitioners. However, 61 (40.7%) of the 150 participants had never received formal child protection training, while 89 (59.3%) reported attending training activities (Table 1).

**Table 1:** Demographic Characteristics of Participants (N=150)

Characteristic	Category	n	%
Profession	Physician	102	68.0
	Nurse	38	25.3
	Other (Allied Health)	10	6.7
Years of Experience	<5	45	30.0
	5–10	58	38.7
	>10	47	31.3
Previous Child Protection Training	Yes	89	59.3
	No	61	40.7

Knowledge assessment

Awareness of child protection laws was generally high. One hundred forty (93.3%) were aware of the Omani Child Law, and 122 (81.3%) correctly identified the legal definition of a child as under 18 years. Regarding reporting mechanisms, 128 (85.3%) knew that a national child protection hotline exists. However, procedural knowledge gaps emerged: while 110 (73.3%) of the total sample knew the correct hotline number (1100), this represented 91 of 128 (71.1%) among those who were aware the hotline existed. This 12-percentage-point gap between awareness and accurate knowledge indicates that general awareness of resources does not guarantee procedural knowledge necessary for effective implementation (Table 2).

**Table 2:** Knowledge of Child Protection Framework (N=150) *Percentages of total sample (n=150) **Among those aware hotline exists (n=128): 91 of 128 = 71.1%

Knowledge Item	Correct Response	n	%
Awareness of Omani Child Law	Yes	140	93.3
Legal definition of child (<18 years)	Yes	122	81.3
Existence of national child protection hotline	Yes	128	85.3
Correct hotline number (1100)*	Yes	110	73.3

Attitudes toward child abuse reporting

The majority of participants, 138 (92.0%), believed that cases of suspected child abuse should always be notified to authorities. However, eight participants (5.3%) reported that notification should occur often, and four participants (2.7%) indicated occasional notification. Regarding the existence of local clinical guidelines for managing suspected child abuse cases presenting to healthcare facilities, 127 (84.7%) were aware that such guidelines exist, whilst 15 (10.0%) stated guidelines do not exist, and eight participants (5.3%) were uncertain (Table 3).

**Table 3:** Attitudes Toward Child Abuse Reporting (N=150)

Statement	Response	n	%
Cases of suspected child abuse should be notified	Always	138	92.0
	Often	8	5.3
	Occasionally	4	2.7
Existence of local clinical guidelines for managing suspected child abuse cases	Yes	127	84.7
	No	15	10.0
	Not sure	8	5.3

Self-reported practices

While 110 of 150 participants (73.3%) reported "always" notifying authorities when abuse was suspected, 138 of 150 (92.0%) believed that reporting should always occur, representing an 18.7-percentage-point difference at the aggregate level. This aggregate-level analysis reflects differences in attitude and practice distributions across the sample rather than paired individual-level analysis of whether specific participants with supportive attitudes also report consistently.

Doctors were identified as responsible for official notification by 139 of 150 participants (92.7%). This concentration of perceived reporting responsibility among physicians may create procedural constraints, although this was not directly assessed in the present study (Table 4).

**Table 4:** Self-Reported Practices in Child Abuse Cases (N=150)

Practice Item	Response Category	n	%
Frequency of reporting when abuse suspected	Always	110	73.3
	Usually	28	18.7
	Sometimes	10	6.7
	Rarely/Never	2	1.3
Primary person responsible for reporting	Doctor	139	92.7
	Nurse	8	5.3
	Other	3	2.0

As contextual information, Al Dhahira Regional Health Directorate records indicated that 60 suspected child abuse cases were officially reported across all healthcare facilities in 2023. However, the present study did not assess the number of cases personally encountered by each participant. While 110 (73.3%) reported they would "always" report, this stated intention exists largely in the theoretical domain, as the actual frequency of case encounters among individual participants remains unmeasured.

Barriers to reporting

Participants were asked to identify the single most commonly faced challenge when notifying child abuse cases. When selecting one primary barrier, fear of legal consequences was identified by 44 (29.3%), clinical identification challenges by 42 (28.0%), lack of feedback about patient outcomes by 39 (26.0%), and lack of identified focal person by 24 (16.0%) (Table 5). Additionally, regarding participation in child protection training activities, 89 (59.3%) reported attending training more than once or once, whilst 61 (40.7%) had never participated in child protection training activities.

**Table 5:** Most Commonly Faced Barriers to Child Abuse Reporting (N=150)

Barrier	n	%
Fear of legal consequences	44	29.3
Inability to clinically identify child abuse cases	42	28.0
Lack of feedback about patient's outcome	39	26.0
Lack of identified focal person	24	16.0

Factors associated with reporting behavior

Further analysis examined factors associated with consistent reporting behavior. Healthcare workers with previous training (n=89) were significantly associated with consistent reporting: 73 (82.0%) reported always reporting compared to 37 (60.7%) among untrained workers (n=61), p=0.001. This significant association indicates that training participation is related to more consistent reporting behavior.

Healthcare workers with 5-10 years of experience demonstrated the highest reporting rates at 46 (79.3%), while those with less than five years experience reported less consistently at 28 (62.2%). Those with more than 10 years of experience reported at 36 (76.6%) (p=0.045).

Physician profession was associated with higher reporting rates compared to nurses and other allied health professionals, though this difference did not reach statistical significance: 80 (78.4%) of physicians reported always, compared to 24 (63.2%) of nurses and 6 (60.0%) of other allied health professionals (p=0.068).

Higher knowledge scores were significantly associated with consistent reporting behavior: 89 (78.8%) for high scores (>80%) versus 21 (56.8%) for low scores (≤80%), p=0.002 (Table 6).

**Table 6:** Factors Associated with "Always" Reporting Suspected Child Abuse (n=150)ᵃ *p<0.05, statistically significant ᵃChi-square test used; Fisher's exact test applied whenever appropriate for categorical variables.

Factor	Category	Always Report n (%)	Inconsistent Reporting, n (%)	p-value
Previous Training	Yes	73 (82.0)	16 (18.0)	0.001*
	No	37 (60.7)	24 (39.3)	
Years of Experience	<5 years	28 (62.2)	17 (37.8)	0.045*
	5–10 years	46 (79.3)	12 (20.7)	
	>10 years	36 (76.6)	11 (23.4)	
Profession	Physician	80 (78.4)	22 (21.6)	0.068
	Nurse	24 (63.2)	14 (36.8)	
	Other	6 (60.0)	4 (40.0)	
Knowledge Score	High (>80%)	89 (78.8)	24 (21.2)	0.002*
	Low (≤80%)	21 (56.8)	16 (43.2)	

## Discussion

Findings in the context of the literature

This first systematic assessment of healthcare workers' knowledge, attitudes, and practices regarding child abuse in Al Dhahira identified a substantial gap between stated attitudes and self-reported practices. Despite widespread endorsement of mandatory reporting policies, actual reporting behavior fell significantly short, a finding that aligns with international research demonstrating that awareness of child protection frameworks does not consistently translate into reporting action [3].

Multiple implementation barriers account for this gap, including fear of legal consequences, clinical identification challenges, and lack of feedback about case outcomes. Of particular concern, the concentration of reporting responsibilities among physicians may create systemic bottlenecks in the reporting pathway, although this was not directly assessed in the present study. A critical distinction emerged between general awareness and procedural competence: while most healthcare workers were aware that a national hotline exists, far fewer possessed accurate knowledge of reporting procedures. Qualitative research has highlighted that primary healthcare professionals often rely on subtle clinical cues and intuitive judgment in identifying suspected abuse, suggesting that recognition involves more than formal knowledge of definitions and procedures [12]. This gap between awareness and actionable knowledge suggests that information campaigns alone are insufficient for effective implementation. Similar patterns have been documented internationally among nurses [6], where reporting intentions are shaped not by awareness alone but by organizational barriers, legal concerns, and feedback mechanisms.

Barriers to reporting

Training participation was significantly associated with consistent reporting behavior. However, given the cross-sectional study design, this association does not establish causation. Alternative explanations merit consideration: reverse causation (healthcare workers who already report consistently may preferentially seek training), self-selection bias (individuals choosing training differ in unmeasured ways), confounding variables (years of experience, institutional culture, individual motivation), and temporal uncertainty (whether training preceded or followed consistent reporting adoption).

The association between training and consistent reporting is noteworthy and suggests that professional development may be one factor among many contributing to reporting behavior. Literature on training outcomes demonstrates associations between training and improved knowledge and reporting behaviors [9]. However, this study's cross-sectional design cannot establish whether training causes improved reporting. Notably, a substantial proportion of healthcare workers had never participated in training, and even among those trained, some reported inconsistent practices. This underscores that training alone is insufficient; comprehensive child protection strategies must address multiple levels simultaneously: individual (training, knowledge), institutional (focal persons, procedures, feedback), and systemic (legal protections, organizational support).

Regional and global context

The attitude-practice gap identified in Al Dhahira is not unique to Oman. A scoping review of child maltreatment and protection across Arab Gulf Cooperation Council countries identified attitudes, awareness, and reporting practices as key research themes, with substantial variations documented across settings [13]. International comparisons reveal markedly different implementation patterns.

Reporting behavior in Al Dhahira's primary healthcare settings substantially exceeded that documented among Saudi physicians, where fewer than one in seven had reported a suspected case in the preceding six months, despite over 90% expressing willingness to report [11]. Australian emergency department staff demonstrated intermediate reporting rates [5]. These differences highlight that the attitude-practice gap is not a universal phenomenon but varies considerably by context.

The magnitude of the discrepancy between attitudes and reported practices also varies substantially by setting. Al Dhahira demonstrated a notable gap between endorsement of mandatory reporting and actual practice; however, this gap was considerably smaller than reported in comparable international contexts [11]. These variations suggest that implementation barriers are highly contextual and that effective interventions must address setting-specific factors rather than applying universal approaches.

Procedural knowledge, particularly awareness of specific reporting mechanisms, also differed markedly across regions. Knowledge of correct reporting procedures was substantially higher among Al Dhahira healthcare workers than among comparable professional groups elsewhere [11]. Barriers to reporting similarly reflected contextual factors: primary obstacles in Al Dhahira included legal concerns, clinical identification challenges, and limited feedback mechanisms. In contrast, international comparisons revealed different barrier profiles, with some settings reporting time constraints and educational gaps as predominant barriers [5,11]. This heterogeneity in both barriers and knowledge levels underscores the necessity of context-specific implementation strategies that address locally relevant obstacles rather than generalized interventions.

Al Dhahira context and implementation science

Al Dhahira's remote location amplifies these findings' importance. When primary healthcare centers serve as the sole medical contact for remote communities, worker competence becomes critically essential, and system failures have greater consequences. Research examining readiness for child maltreatment prevention program implementation in Oman identified that the country demonstrates high readiness in legislation and policy but significant gaps in human resource capacity and implementation [8]. 

The Sultanate of Oman has made substantial progress in developing child protection system capacities [7] and has committed to a public health approach benchmarking against international standards such as the INSPIRE Strategy for comprehensive child protection [10]. This strategic framework provides a foundation for multi-level interventions targeting individual (healthcare worker training), organizational (institutional support and governance), and system factors (policies, procedures, coordination) simultaneously.

Implications for intervention design

The attitude-practice gap identified in this study is not unique to Al Dhahira but reflects systemic challenges throughout healthcare systems [13]. Addressing this gap requires coordinated, evidence-based approaches warranting prospective evaluation, potentially including professional development programs using interactive techniques; clear communication about legal protections and reporting procedures; establishment of designated child protection focal persons within healthcare facilities; implementation of feedback mechanisms to inform reporters about case outcomes; and strengthening institutional governance structures to support healthcare workers in their child protection role.

The significant association between training and consistent reporting behavior suggests that professional development warrants further investigation as a potential intervention. However, prospective longitudinal evaluation with control groups and objective outcome measures is needed to establish whether such interventions produce sustained behavioral change. Training must be coupled with organizational and system changes that enable healthcare workers to translate their knowledge and commitment into consistent protective action.

Limitations

This cross-sectional study has several important limitations. First, the study design allows establishment of associations between variables but cannot determine causal relationships. The observed association between prior training and consistent reporting behavior may reflect reverse causation (workers who already report consistently may preferentially seek training), selection bias (more conscientious workers may both report more and seek training), or confounding variables. Prospective longitudinal designs with control groups would be needed to establish whether training interventions actually cause improved reporting behavior.

Second, data collection via online questionnaire introduces potential social desirability bias. Participants may report what they believe they should do rather than actual practice, particularly regarding child abuse reporting-a topic with strong ethical and legal imperatives. This may partially explain the 18.7-percentage-point gap between stated attitudes and reported practices. Additionally, the assessment relied on self-reported knowledge and practices rather than directly observing healthcare worker behavior or reviewing medical records for actual reporting data. Self-reported practice data may be subject to bias in either direction, depending on individual perspectives and recall accuracy.

Third, the questionnaire used dichotomous categorization of attitudes (supportive/non-supportive), practices (consistent/inconsistent), and knowledge (high/low at >80%) rather than continuous measurement. While this categorical approach provides clinically meaningful distinctions aligned with mandatory reporting obligations, it may not capture nuanced variations across the full spectrum of knowledge acquisition, attitude strength, or reporting frequency. Continuous or multi-level categorical measures might provide a more granular understanding of variation.

Fourth, although the questionnaire collected both attitude and practice responses from each participant, the analysis did not include individual-level paired analysis to examine whether specific participants with supportive attitudes also report consistently. Aggregate-level differences are reported but cannot definitively characterize whether the gap primarily reflects participants with supportive attitudes but inconsistent practices, or differences in attitude distribution across the sample. Future studies should explicitly design for paired individual-level cross-tabulation analysis to strengthen understanding of attitude-practice consistency.

Finally, while findings are limited to Al Dhahira region, they may be relevant to other Omani regions given the standardized healthcare system and unified child protection frameworks. However, international generalizability is limited by differences in legal frameworks, healthcare systems, and cultural contexts that may affect applicability.

Despite these limitations, this is the first systematic assessment of healthcare worker knowledge, attitudes, and practices regarding child abuse in Al Dhahira's remote primary healthcare setting, addressing a critical evidence gap.

## Conclusions

Healthcare workers in Al Dhahira demonstrate good theoretical knowledge of child protection frameworks and strong attitudes supporting reporting obligations yet face significant practical barriers compromising consistent implementation. An 18.7-percentage-point attitude-practice gap exists, influenced by multiple factors including fear of legal consequences, clinical identification challenges, lack of feedback, and organizational barriers. Training participation was significantly associated with more consistent reporting behavior; however, given the cross-sectional study design, this association does not establish causation and may reflect self-selection bias or confounding variables.

Evidence-based interventions including professional development programs, clear communication regarding legal protections for reporters, establishment of designated child protection focal persons, feedback mechanisms, and development of multi-professional reporting capacity warrant prospective evaluation to bridge the attitude-practice gap. Such evaluation must employ longitudinal designs with objective outcome measures and comparison groups rather than relying on self-reported practices alone. This study provides an important foundation for understanding implementation barriers in primary healthcare and highlights critical gaps that must be addressed through coordinated, evidence-based approaches grounded in implementation science principles.

## Figures and Tables

**Figure 1:**
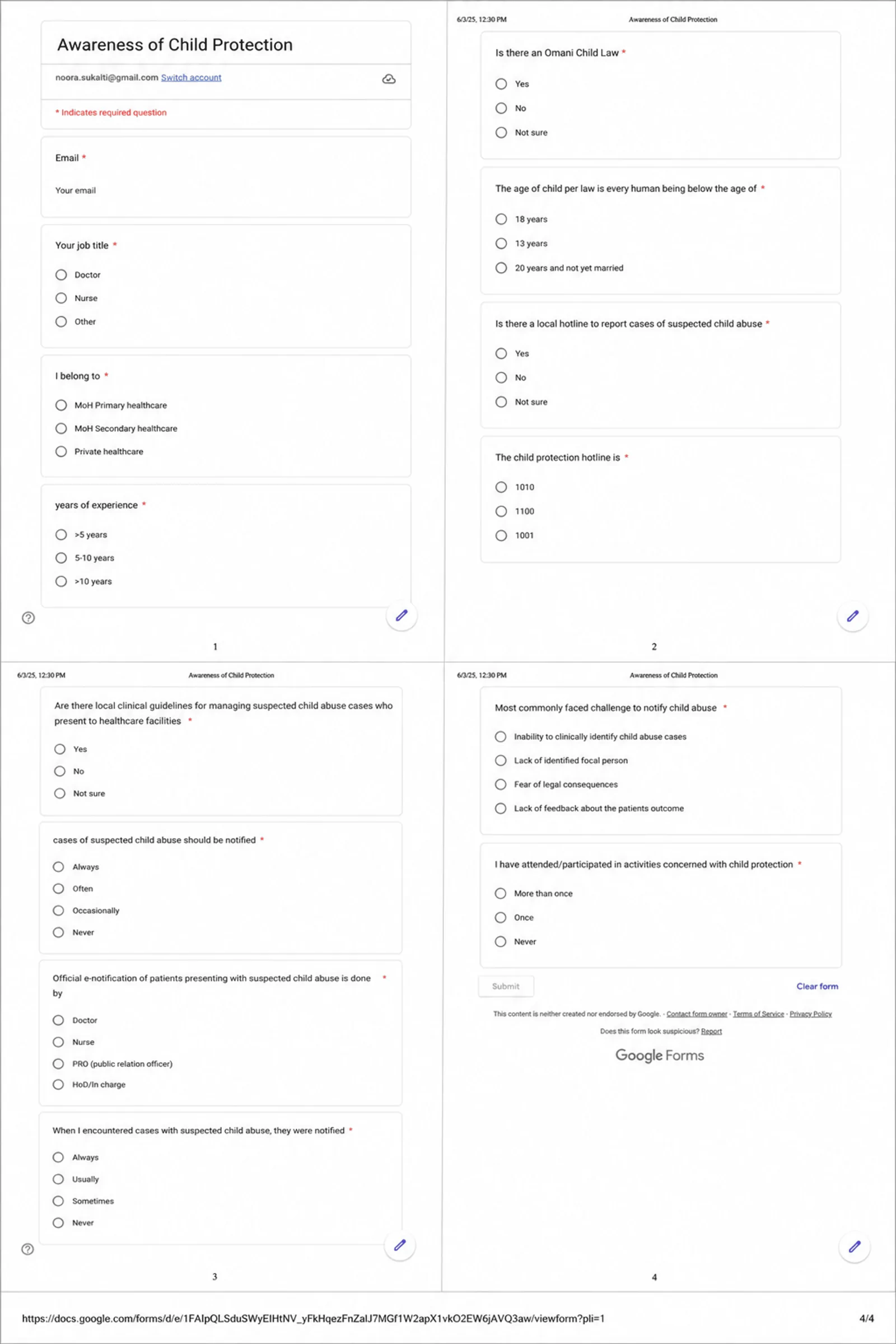
Questionnaire
